# Endoscopic Image Enhancement: Wavelet Transform and Guided Filter Decomposition-Based Fusion Approach

**DOI:** 10.3390/jimaging10010028

**Published:** 2024-01-20

**Authors:** Shiva Moghtaderi, Omid Yaghoobian, Khan A. Wahid, Kiven Erique Lukong

**Affiliations:** 1 Department of Electrical and Computer Engineering, University of Saskatchewan, Saskatoon, SK S7N 5A9, Canada; hwe298@mail.usask.ca (O.Y.); khan.wahid@usask.ca (K.A.W.); 2 Department of Biochemistry, Microbiology and Immunology, University of Saskatchewan, Saskatoon, SK S7N 5E5, Canada; kiven.lukong@usask.ca

**Keywords:** endoscopic images, image enhancement, image fusion, biomedical image processing

## Abstract

Endoscopies are helpful for examining internal organs, including the gastrointestinal tract. The endoscope device consists of a flexible tube to which a camera and light source are attached. The diagnostic process heavily depends on the quality of the endoscopic images. That is why the visual quality of endoscopic images has a significant effect on patient care, medical decision-making, and the efficiency of endoscopic treatments. In this study, we propose an endoscopic image enhancement technique based on image fusion. Our method aims to improve the visual quality of endoscopic images by first generating multiple sub images from the single input image which are complementary to one another in terms of local and global contrast. Then, each sub layer is subjected to a novel wavelet transform and guided filter-based decomposition technique. To generate the final improved image, appropriate fusion rules are utilized at the end. A set of upper gastrointestinal tract endoscopic images were put to the test in studies to confirm the efficacy of our strategy. Both qualitative and quantitative analyses show that the proposed framework performs better than some of the state-of-the-art algorithms.

## 1. Introduction

Endoscopy is a nonsurgical medical procedure for inspecting the structure of tissue and lesions of human digestive tracts with high accuracy [1]. Physicians use endoscopy techniques in different parts of the body such as esophagus, stomach, and colon to diagnose gastrointestinal bleeding, inflammatory diseases, and polyps [2]. Endoscopy is performed with a flexible tube that has a LED light source and camera connected to it [3]. On a monitor, the doctor has access to images of the gastrointestinal system. In an upper endoscopy, an endoscope is smoothly inserted through the mouth into the esophagus. Likewise, endoscopes can also go through the rectum into the colon to examine the lower gastrointestinal (GI) tract.

Endoscopic image visual quality is an important aspect in early lesion detection and surgical treatments. This approach, however, has some limitations that may adversely affect the examination and diagnosing process. Inadequate brightness and contrast and blurred details might result from poor camera quality and inconsistent lighting from the single illumination source [4,5]. Furthermore, endoscopic images may sometimes have bright reflections on a mucus layer. This may cause the imaging performance to drastically decline [6]. The situation deteriorates with capsule endoscopy, primarily due to constraints in power and the capsule’s volume [7]. Thus, some image processing techniques must be used to endoscopic images in order to highlight the details and important features for ease of study in clinical settings [8,9].

To enhance the quality of medical images, numerous image enhancement techniques have been proposed. One popular approach is image fusion, which is described as the process of improving an image’s resolution by combining numerous copies of the image with previously recorded data that are notably distinct from one another [10]. In the domains of image processing and computer vision, multi-exposure image fusion is becoming a prominent area of study because it can merge images with different exposure levels into a high-quality full exposure image [11]. From several images with various exposure settings, multi-exposure image fusion seeks to create an image with the most beneficial visual information. These approaches usually called HDR (high dynamic range) techniques which involve capturing multiple images of the same scene at different exposure levels. Typically, HDR techniques include taking at least three photos: one underexposed (capturing details in bright areas), one overexposed (capturing details in dark areas), and one properly exposed. These images are then merged or combined using specialized software or techniques to create a single high-quality image that contains a broader range of tones, colors, and details [12]. Xu et al. presented a new technique for fusing multiple exposure images based on the tensor product and tensor singular value decomposition [13]. Tensor products and t-Svd are used to create a new fusion technique. In [14], the enhanced weighted, guided filtering algorithm is utilized to enhance tissue visualization in endoscopic images. Endoscopic images of vessels were improved by enhancing vessel features and contours using an unsharp mask algorithm and an improved weighted guided filter. Furthermore, Tan et al. suggested an algorithm for improving endoscopic images that decomposes the input image into a detail layer and a base layer based on noise reduction [15]. In the detail layer, the blood vessel data are channel-extended, and in the base layer, adaptive brightness correction is used. Finally, fusion is performed to obtain the improved endoscopic image. Wang and colleagues [16] suggest a technique for enhancing image’s uniformity and luminance while reducing their overexposure. The suggested technique generates an adaptable brightness weighting that can be applied to improve the luminance of the endoscopic image. In a 2018 study, Xia et al. proposed an image-enhancing technique for endoscopic images with effective noise suppression capability [17]. The illumination and detail layers are each treated individually by the algorithm after it has initially identified the various illumination zones.

The endoscopic image enhancement method based on histogram equalization and unsharp masking in the wavelet domain has been reported [18]. It can disclose details of endoscopic images with poor light. The method is a logarithm-based histogram equalization approach that adjusts the low-frequency wavelet components to improve contrast and prevent artifacts.

In this work, our goal is to improve the endoscopic image quality for ease of study in clinical applications. To do so, three image correction methods are used to split source images into several sub images. Finally, the fusion technique aids in the manipulation of image contrast, which improves image visual quality.

The primary contributions of this paper are outlined as follows:

- We propose an approach to improve the visual quality of endoscopic images by taking advantage of artificially generated sub images and image fusion techniques. We combine three key enhancement methods: detail enhancing, CLAHE, and image brightening.
- Multi-layer wavelet transform and guided filter-based decomposition schemes, which decompose each intensity layer into four coefficients, have been introduced.
- A weighted fusion rule based on local contrast and local entropy is proposed to fuse high-frequency components.

The paper is organized as follows: Our algorithm’s design is presented in Section 2 of the paper; the experimental findings are shown in Section 3 along with a discussion of how well the suggested method works in Section 4. Future works is reported in Section 5. Section 6 describes the conclusion.

## 2. Materials and Methods

This work proposes an endoscopic image enhancement technique based on artificially generated sub-images and fusion schemes. It is worth mentioning that we took advantage of the HIS color space in our work. The HSI (hue, saturation, and intensity) color space, a three-dimensional model that represents colors based on their hue, saturation, and intensity components, has been used. This technique uniquely separates color information from brightness, allowing independent adjustment of color and intensity, which proves beneficial in image processing tasks. This color space’s ability to maintain the original color information while enhancing image features makes it a preferred choice for preserving color fidelity in various applications, aligning well with human visual perception and aiding accurate analysis in fields such as endoscopic imaging [19].

**Figure 1:**
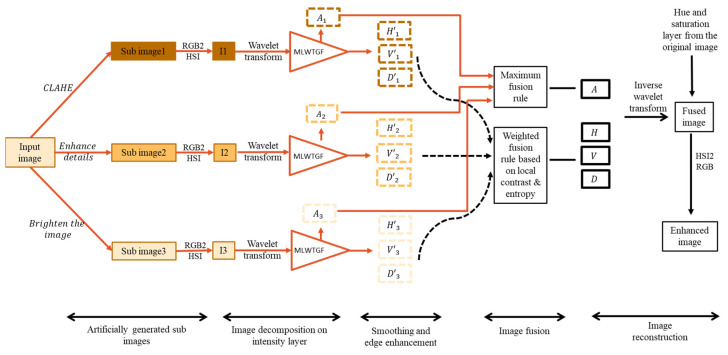
Framework of the proposed image enhancing model.

A framework of the proposed model is illustrated in Figure 1. Three image correction methods are used to split source images into several sub-images. Finally, the fusion technique aids in the manipulation of image contrast, which improves the visual quality of the image. This section covers the proposed method’s comprehensive description.

### 2.1. Generating Sub Images

Limited contrast, limited visibility, low dynamic range, and low signal-to-noise ratio are all characteristics of low-light images. Additionally, the true color of the target cannot be captured because the entire image is underexposed [20]. By first creating three sub images with different characteristics, we attempted to begin the image enhancing process. Sub images are different versions of the original input image which are generated using three image enhancement methods.

Among all multi exposure image fusion methods that have been developed in recent years [11,21], a common technique is to use gamma correction to create the multi exposure derived images as the generated sub images.

Gamma correction is a nonlinear operation on the input image that results in an exponential relationship between the gray values of the output image and the input image [22]. In other words, Gamma correction is used to modify the overall image intensity.

**Figure 2:**
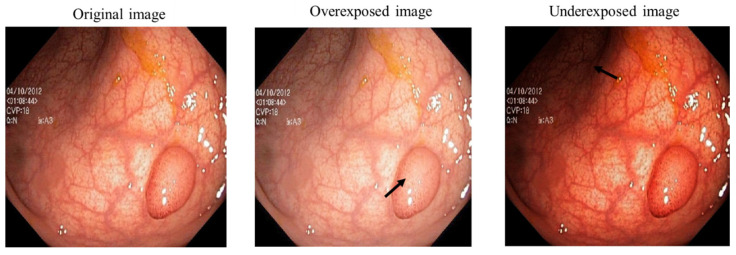
Three generated sub images by gamma correction.

Gamma correction alters the overall image intensity by transforming the power function indicated as Ɣ. As can be seen in Figure 2, when Ɣ < 1, brighter intensities are compressed and the details in highlights are highlighted while Ɣ > 1 highlights the details in shadows.

That is why researchers show interest in low-light image improvement using gamma corrections and adjusting the reflected light on the object surface [23]. However, Gamma corrections may cause some problems as well. For example, as the light increases, some underexposed areas become visible, but areas that were previously well-exposed/overexposed deteriorate because of global exposure adjustments [20]. To solve this issue, we perform three image enhancement methods on the original image to generate three different versions of our input image. By utilizing these methods, we aim to improve the contrast and enhance all the details in the dark and bright regions. This is mainly performed to have an even illumination at the end of the enhancement process.

**Figure 3:**
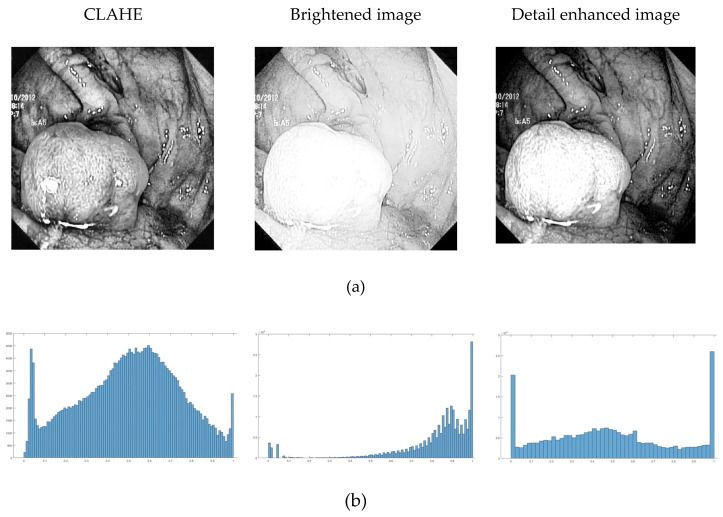
(**a**) The intensity layer of three generated sub images by CLAHE [24], brightened image [25,26,27] and detail enhanced image [28]. (**b**) Their corresponding histogram.

To improve quality, we tried to generate three sub images which are complementary to one another. We used detail enhancement, contrast-limited adaptive histogram equalization algorithm (CLAHE) and the brightened image to generate sub images from a single input image. These three sub images are illustrated in Figure 3. We have also included the histogram demonstration of these sub images to help compare the general contrast and pixel distribution of the images.

To perform adaptive histogram equalization, CLAHE is used to generate the first sub image. CLAHE is based on breaking down the image into several almost equal-sized, non-overlapping areas and performing histogram equalization on each patch [24]. This algorithm improved the local contrast of bright spots. To enhance the features of the dark areas, we have used image brightening to improve the contrast in darker areas and generally enhance the image’s contrast as our second sub image for this work. This is mainly performed based on an objective function which consists of image entropy [25,26,27]. The third sub image is retrieved using local Laplacian filtering. It uses straightforward processing to alter the image in an edge-aware manner [28].

Unlike HDR techniques, our approach does not rely on capturing multiple exposures of the same scene; instead, it works with the single input image using a combination of techniques.

### 2.2. Image Decomposition Based on Multi Level Wavelet Transform and Guided Image Filtering (MLWTGF)

The source image is divided into multiple sub images. The following step is to decompose these three images into explanatory layers. One mathematical technique that has gained growing prominence for efficiently extracting image’s information is the wavelet transform [29]. By applying image decomposition based on wavelet transform theory, it is possible to extract an image’s information relating to the horizontal, vertical, and diagonal directions. The coefficients resulted from the wavelet transform are LL, LH, HL, and HH. The source image’s approximation coefficient is represented by LL while others are detail coefficients [30].

**Figure 4:**
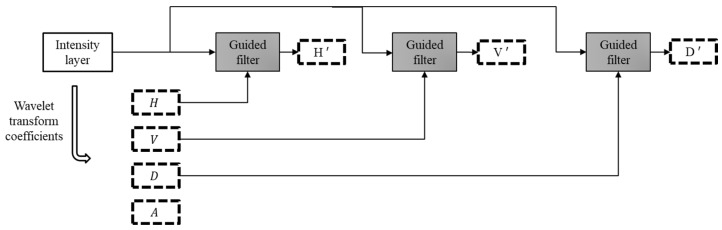
Block diagram of the proposed decomposition scheme.

We then use the coefficients as the guidance image for guided filter to enhance the edges and structural information. The block diagram of the proposed decomposition scheme is demonstrated in Figure 4. The intensity layer of each input image is enhanced through guided filter.

**Figure 5:**
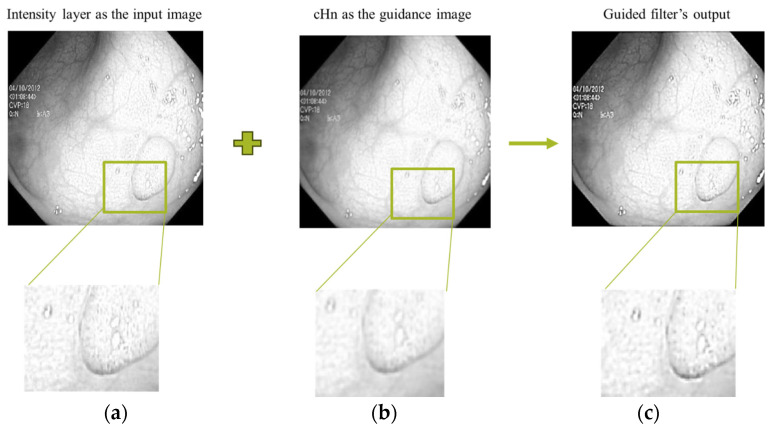
(**a**) Intensity layer as the input image. (**b**) Horizontal detail coefficient as the guidance image. (**c**) Guided filter’s output.

An example output image of the guided filter using our proposed decomposition scheme is shown in Figure 5. We have used the detail coefficients as the guidance image. The detail coefficient is resulted from the wavelet transform. The goal is to efficiently transfer the structure details to the resulted filtered image. It can be seen in the intensity layer and filtered sub images that significant horizontal, vertical, and diagonal features are effectively transferred from the corresponding guidance filter (cHn).

### 2.3. Image Fusion

Employing the above-mentioned decomposition approach, the sub layer containing rich structural details (LH, HL, and HH) and background information (LL) are generated. The proper fusion rules should be applied on the captured components from three input images. Based on the component’s characteristics, fusion strategy should be selected. Most of the approximation information (the background) from the input images is presented in the LL components, which is captured from the low frequency layers. Thus, the maximum-value fusion approach is applied to make sure that more texture-related features are preserved (Equation (1)).

(1) $$A_{Fused}=Max(A_{1},A_{2}{,A}_{3})$$

Detail components contain the edge, corner, and structure information of the input source images. A weighted fusion rule is chosen to fuse high frequency components. In weighted fusion methods, the coefficients of different local areas are given varying weights [31]. Weights denoting the relative significance of each combined image.

The choice of weight is fundamental since it directly affects the fused image. Selecting an unsuitable weight will result in unstable algorithm performance [32]. We have considered two parameters for weighing function: local contrast and local entropy. In a 3 × 3 neighborhood, local contrast calculations will be made between the centered cell and the surrounding cells to determine the local contrast information [33]. In other words, local contrast measures that the pixel is variable form the surrounding pixels. On the other hand, local entropy is a metric for information density [34]. The input image’s texture can be described using entropy, a statistical indicator of randomness [35].

For each 3 × 3 neighborhood in the fusion input images, we obtained the local contrast and local entropy. The regional characteristics provide a quantitative analysis of pixel intensity swings in an image. At this point, we allocate weights to the fusion’s input images. In general, a larger weight should be given to the patch with more details and better contrast. The weights are assigned based on prioritized local contrast and local entropy. We can control the trade-off between contrast and entropy by modifying the weighting parameters, resulting in the fused image having the desired level of detail preservation and contrast enhancement. To prioritize detail preservation, we have given higher weights to local entropy.

The weighing criteria for fusing two detail components based on local contrast and local entropy are formulated as follows:

(2) $$W_{IA}=\gamma_{1}.C_{A}+\gamma_{2}.E_{A}$$

(3) $$W_{IB}=\gamma_{1}.C_{B}+\gamma_{2}.E_{B}$$

where γ_1 and γ_2 indicate the weighting parameters. The fused sub image is obtained by the weighted fusion approach:

(4) $$I_{Fused}=\sum_{i}^{n}(W_{IA}\left(I_{A}\right)+W_{IB}\left(I_{B}\right))$$

After extracting the four fused components, we perform the inverse wavelet transform to generate the final enhanced image.

## 3. Results

We examined our architecture using a readily available endoscopic image collection of the gastrointestinal tract. The open-access Kvasir dataset contains images of the GI tract that highlight anatomical landmarks and pathological findings [36].

We evaluated how effective our suggested framework performed in this section. Our methodology has been compared to four other image enhancement techniques. Comparison strategies include enhancing method for weakly illuminated images [37], endoscopic image luminance enhancement [16], enhancement method for correcting low-illumination images [38], and LIME [39]. All these papers use the same approach to use different sub images of the input image.

All other related enhanced images of four different approaches are developed by publicly available codes. All the experiments are run in MATLAB (R2023a) on an 11th Gen Intel(R) Core(TM) i7, 3.00 GHz and 16.0 GB RAM computer.

To assess the method’s efficiency, we conduct subjective and objective assessments in our experiments. Furthermore, to evaluate how applicable our method is, we have designed a scoring system. The doctors were asked to grade the images on a scale of 1 to 5 (1: Poor/2: Average/3: Good/4: Very good/5: Excellent).

### 3.1. Qualitative Analysis

**Figure 6:**
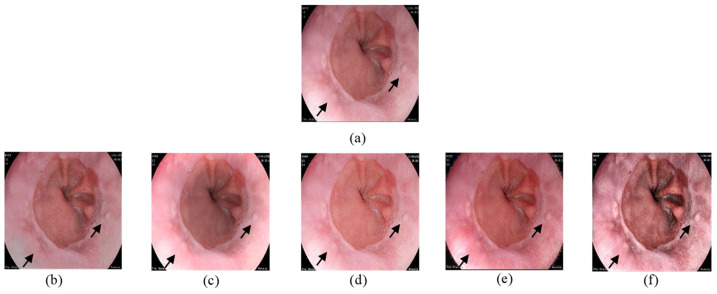
Comparison of enhanced images from Kvasir dataset. (**a**) The input image; (**b**) [37]; (**c**) [16]; (**d**) [38]; (**e**) [39]; (**f**) proposed.

Physicians mostly use endoscopic images to analyze and interpret images of artery walls and organ tissues gathered from patients [15]. That is why visual comparison of improved images is essential. This section reports the image enhancement results when compared to other methods. In Figure 6, the input image demonstrates the Z line between the esophagus and the stomach. We have tried to enhance the input image’s visualization with 5 different methods.

As can be seen, there appears to be lack of contrast in Figure 6b–d and an improvement in the brightness and clarity in general, but some information is lost especially in brighter areas. It can be verified that our suggested strategy is more effective than the previous publications in terms of improving visual quality and highlighting details. The proposed enhancing strategy improves image contrast in the normal brightness area, while the details are highlighted in the dark section as well. Also, the output images show no signs of noise, over enhancing or color distortion. This demonstrates that our recommended algorithm is appropriate for low-light image enhancing applications.

**Figure 7:**
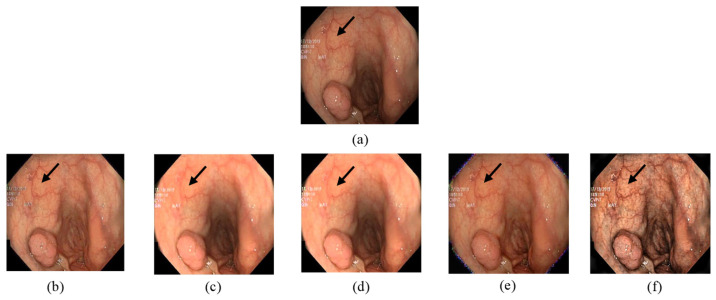
Comparison of enhanced images from Kvasir dataset: (**a**) the input image; (**b**) [37]; (**c**) [16]; (**d**) [38]; (**e**) [39]; (**f**) proposed.

In Figure 7, the input image contains a polyp and blood vessels. The enhanced image must improve the general contrast while emphasizing the vessels details to fit the observer’s normal perceptive spectrum. The outputs in Figure 7b,e clearly have a better demonstration in darker areas. On the other hand, over enhancement happened with Figure 7c,d. The brightness of lighter regions is improved in a way that blood vessel information is lost. In Figure 7f, our proposed method’s output increased the visibility in darker areas and enhanced details in all regions.

**Figure 8:**
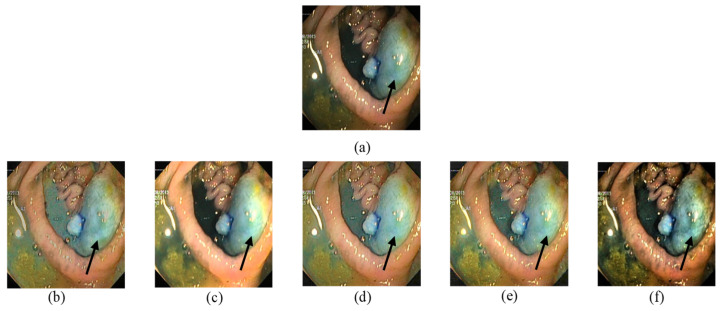
Comparison of enhanced images from Kvasir dataset: (**a**) the input image; (**b**) [37]; (**c**) [16]; (**d**) [38]; (**e**) [39]; (**f**) proposed.

Figure 8 also illustrates improved image visualization in Figure 8f with enhanced detail and overall contrast.

In order to enhance our evaluation, we contacted two skilled medical professionals who regularly perform endoscopy procedures. The physicians were given a collection of images, including those produced by our proposed method as well as algorithms from other researchers. The doctors were asked to grade the images on a scale of 1 to 5 (1: Poor/2: Average/3: Good/4: Very good/5: Excellent). The average ratings given by human observers are related to ten test images and are shown in Table 1. All output images are provided in Supplementary File. Our method’s outputs gain higher scores in comparison with the other four methods. In general, our suggested enhancement strategy improved the visual contrast and earned a favorable subjective evaluation by the professional observers. This is consistent with the claim that our provided enhancing algorithm can improve the general contrast and enhancing the details.

### 3.2. Quantitative Analysis

In the following section, we will compare the effects of the proposed strategy to existing ways using evaluation metrics. There are two primary methods for providing an objective evaluation of an image enhancement approach: First is the full-referenced image quality metric which considers information from both the modified image and a reference image. The second is no-referenced image quality metrics. These indexes attempt to estimate perceptual quality only based on the output image [40]. However, due to lack of a perfect reference image, it is a challenging task for many computer vision scenarios [41]. To illustrate the effectiveness of our method, six indexes have been selected from both categories.

Entropy: determines the fused image’s texture information.

CII (contrast improvement index): measures the extent of enhancement of contrast before and after image processing [5].

PIQE (perception-based image quality evaluator): the no-reference image quality metric, which has an inverse relationship with an image’s perceived quality [42].

PCQI (patch-based contrast quality): estimates the image’s overall contrast quality while simultaneously constructing a quality map that has the local changes [43].

PSNR (peak signal-to-noise ratio): a byte-by-byte comparison of the two images without considering what they actually represent, hence it can only approximate the image quality as perceived by human observers [44]. The difference between the image before and after processing is reflected in the PSNR. The difference becomes smaller as the PSNR value increases.

SSIM (structural similarity index): with SSIM, two image’s similarity can be calculated based on brightness and the contrast [45].

In this section, the tables display the results of an objective evaluation of ten images that were enhanced using various techniques. The top two results are highlighted in bold. Table 2 reports that our suggested algorithm and [39] have relatively higher entropy than other methods. This confirms that these two methods can enhance visual contrasts and provide information about the distribution of pixel intensities. In Table 3, we have compared the CII to measure the extent of enhancement of contrast before and after image processing. As can be seen our proposed method shows significant contrast improvement that can compensate the effects of poor camera quality and inconsistent lighting from the single illumination source.

Table 4 represents another no-reference image quality metric PIQE, which places an emphasis on perceptual quality evaluation. Our method and [37] have the top two results among the 10 test images. This supports these two method’s abilities to produce improved visual experiences. Also, PCQI, a strong patch-based index, demonstrating the method’s capability for perceptually transforming the image’s information is demonstrated in Table 5.

We have also reported the comparison results for two full-reference image quality measurements. For Table 6 and Table 7, we have considered the input image as the reference image. However, this may not be the best effort to evaluate the enhancement efficiency, but it is a common practice since reference image is not available. The outputs generated with our method have higher PSNR which means better visuality of the reconstructed image. SSIM is also presented as the original image and the enhanced images similarity based on brightness and contrast. Methods [16,37] and ours have relatively better SSIM values.

## 4. Discussion

To summarize, in this section, we have reported the comparison results between our proposed methods and other image enhancing approaches. While other image enhancement methods have shown promising results in image enhancement techniques, there are still some limitations in terms of local contrast, detail preservation, and applicability for medical practitioners. To address these issues, this article suggests an alternative approach which consists of three sections: The first part is image decomposition based on wavelet transform and guided filter that decomposes the input image while maintaining the details of the input image.

**Figure 9:**
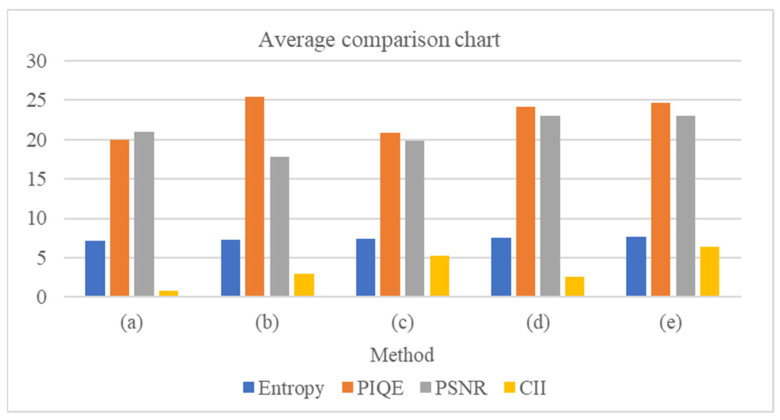
Average comparison chart. Each bar corresponds to the average value a specific metric of 10 images: (a) [37], (b) [16], (c) [38], (d) [39], (e) Proposed.

Second is image fusion that combines different characteristics of image’s sub layers and finally the image reconstruction that includes inverse wavelet transform. Figure 9 reports the average value a specific metric of 10 images to have a better understanding of the results. The outputs generated with our method have relatively better performance. Overall, the findings demonstrate that our suggested methodology performs better than the other papers. The suggested method has an acceptable enhancement effect that raises the brightness of dark objects, improving the clarity and color, and making the images more congruent with human vision which is advantageous to the diagnosing procedure.

It is worth mentioning that the inherent subjectivity in the process of image enhancement should be acknowledged. Factors such as endoscopy’s device illumination and imaging technology play important roles in the original endoscopic image’s quality. We recognize that the interpretation of ‘best images’ can be subjective and influenced by individual expertise. However, we tried to report a detailed description of our work. The paper’s focus is on increasing the visual quality of endoscopic images by taking advantage of artificially generated sub images using three key well known enhancement methods and performing image fusion techniques. Our suggested method consists of three main stages that have been explained with detailed description that facilitates the reproducibility of our results, aiming to enhance the applicability of our method across different clinical settings.

## 5. Future Work

As a future work, we can utilize the improved images generated by our algorithm for the detection and segmentation of various abnormalities, such as polyps, in gastrointestinal (GI) tract endoscopic images. Since medical images often suffer from low contrast and blurred details, it is always challenging to distinguish between different structures. Techniques like ours can perform image enhancement as a preprocessing step by tackling common problems related to endoscopic images. Preprocessing is a pivotal step in medical image processing applications, such as image segmentation and classification. For example, segmentation techniques seek to precisely distinguish the border of the polyp from the surrounding tissue in addition to detecting polyps. Monitoring the resulting polyp segmentations validates that the least favorable segmentation outcomes are linked to lower quality input images or relatively harder-to-identify polyps [46,47]. In such cases, our reported algorithm can ensure that the input raw data are optimized for subsequent analysis. It may lead to more accurate and reliable results in the identification of regions of interest or abnormalities.

## 6. Conclusions

In this study, we introduce a method for enhancing endoscopic images. The first step is to generate three derived sub images from the single input image which are complementary to one another in terms of local and global contrast. By utilizing CLAHE, image brightening, and detail enhancing methods, we tried to generate complementary sub images. We then used a novel multi-level wavelet transform and guided filter-based decomposition technique to decompose each sub layer. The necessary weighted fusion rules are then applied at the end to produce the final improved image. The suggested technique increases the brightness of dark objects while enhancing their clarity and color, which is an acceptable enhancement effect. The proposed enhancing strategy improves image’s contrast in the normal brightness area, while the details are highlighted in the dark section as well. Also, the output images show no signs of noise, over enhancing, or color distortion. This demonstrates that our proposed strategy is appropriate for low-light image enhancing applications.

## Figures and Tables

**Table 1:** The average ratings given by observers.

Input Image	[37]	[16]	[38]	[39]	Proposed
Image 1	2	3	3.5	4.5	4.5
Image 2	2.5	3	4.5	4.5	5
Image 3	2	3	3.5	5	3.5
Image 4	2.5	3	4	4	4.5
Image 5	2	3.5	4	4	4.5
Image 6	2.5	3	3	3.5	4.5
Image 7	2	2.5	3	4.5	4.5
Image 8	2.5	3	3	4	4.5
Image 9	2	2	3	4	4.5
Image 10	2.5	2.5	2.5	4	4.5

**Table 2:** The entropy outcomes from various methods.

Input Image	[37]	[16]	[38]	[39]	Proposed
Image 1	7.1833	7.4410	7.4825	**7.7052**	**7.8653**
Image 2	7.3407	**7.6590**	7.5401	7.6233	**7.7428**
Image 3	7.1878	**7.6534**	7.4041	7.4818	**7.7089**
Image 4	6.9265	7.1816	6.9851	**7.2838**	**7.7392**
Image 5	7.0741	7.6059	7.4964	**7.6235**	**7.7031**
Image 6	7.6242	7.5985	**7.7606**	7.7377	**7.7544**
Image 7	7.2864	7.0792	7.2658	**7.5430**	**7.5234**
Image 8	7.2788	6.8253	7.4715	**7.7203**	**7.7822**
Image 9	6.9022	6.9431	7.1045	**7.3771**	**7.6132**
Image 10	7.1645	7.1092	7.3451	**7.6629**	**7.6463**

**Table 3:** The CII outcomes from various methods.

Input Image	[37]	[16]	[38]	[39]	Proposed
Image 1	0.9878	2.0399	**5.4268**	4.2233	**6.9864**
Image 2	0.8584	3.0497	**4.3574**	**5.6060**	4.0511
Image 3	0.6856	5.3032	**10.6064**	**8.60289**	3.2836
Image 4	0.9326	5.1778	5.0201	**8.0599**	**10.0784**
Image 5	0.5990	2.5144	4.7873	**4.9990**	**5.1079**
Image 6	0.8387	**2.4099**	1.4605	1.9431	**5.0698**
Image 7	0.9727	1.4867	**3.9971**	2.7027	**7.9269**
Image 8	0.9443	2.7898	2.5004	**2.7900**	**5.7693**
Image 9	0.76643	2.4535	**10.857**	**8.5560**	4.3330
Image 10	0.8912	2.7751	3.7644	**4.6609**	**11.8316**

**Table 4:** The PIQE outcomes from various methods.

Input Image	[37]	[16]	[38]	[39]	Proposed
Image 1	42.1150	**25.2324**	40.8786	51.2644	**37.4767**
Image 2	19.4745	**17.2627**	**18.9662**	24.2900	29.8537
Image 3	14.9021	**11.7482**	**13.7531**	17.5451	16.3434
Image 4	**14.2059**	24.7821	15.3968	**14.6998**	31.4279
Image 5	**13.6932**	26.7682	15.9563	15.5255	18.3837
Image 6	**19.8028**	29.0356	23.9177	24.0370	**18.5096**
Image 7	30.2362	39.1689	**29.7034**	34.5068	**25.7705**
Image 8	**20.1907**	34.4065	24.9211	25.7896	**24.0150**
Image 9	**6.9389**	20.1736	8.18611	**8.1098**	19.7488
Image 10	17.9276	25.3987	16.7112	**25.8942**	**24.7609**

**Table 5:** The PCQI outcomes from various methods.

Input Image	[37]	[16]	[38]	[39]	Proposed
Image 1	0.9945	0.9992	**0.9997**	**0.9994**	0.9991
Image 2	0.9943	0.9988	**0.9996**	**0.9989**	0.9985
Image 3	0.9948	0.9990	**0.9991**	**0.9996**	0.9990
Image 4	0.9953	0.9993	0.9988	**0.9994**	**0.9996**
Image 5	0.9954	0.9992	**0.9994**	**0.9997**	0.9986
Image 6	0.9936	0.9981	**0.9988**	0.9986	**0.9987**
Image 7	0.9950	0.9983	0.9986	**0.9992**	**0.9989**
Image 8	0.9946	0.9989	**0.9994**	**0.9993**	**0.9993**
Image 9	0.9953	0.9991	0.9994	**0.9998**	**0.9989**
Image 10	0.9958	**0.9987**	0.9948	0.9973	**0.9994**

**Table 6:** The PSNR outcomes from various methods.

Input Image	[37]	[16]	[38]	[39]	Proposed
Image 1	22.27	18.97	19.94	**24.06**	**22.78**
Image 2	**20.19**	17.47	19.75	19.22	**23.72**
Image 3	19.51	17.73	20.03	**23.72**	**24.00**
Image 4	**26.84**	18.09	17.68	**26.06**	22.15
Image 5	19.17	19.63	22.49	**23.05**	**25.44**
Image 6	17.41	14.09	**18.22**	17.75	**22.59**
Image 7	19.29	14.27	16.44	**21.75**	**19.87**
Image 8	21.11	17.44	21.54	**21.86**	**23.12**
Image 9	22.82	19.56	23.04	**27.60**	**25.19**
Image 10	20.98	21.36	19.21	**25.09**	**21.79**

**Table 7:** The SSIM outcomes from various methods.

Input Image	[37]	[16]	[38]	[39]	Proposed
Image 1	0.9809	**0.9541**	0.9479	0.9271	**0.9848**
Image 2	**0.9258**	**0.9327**	0.9219	0.8803	0.9083
Image 3	**0.9304**	**0.9431**	0.9329	0.9281	0.9136
Image 4	**0.9848**	0.9511	0.9398	**0.9402**	0.9072
Image 5	0.9348	**0.9663**	**0.9561**	0.9036	0.9391
Image 6	**0.8403**	0.7902	0.8359	0.8010	**0.8967**
Image 7	**0.9071**	0.8648	0.8552	0.8709	**0.9032**
Image 8	**0.9522**	0.9155	**0.9358**	0.8730	0.9042
Image 9	0.9508	**0.9605**	0.9540	0.9603	**0.9525**
Image 10	**0.9489**	**0.9732**	0.9418	0.9319	0.9273

## Data Availability

We have made the code publicly available on: https://github.com/S-M-Cloud/Endoscopic-Image-Enhancement-Wavelet-Transform-and-Guided-Filter-Decomposition-Based-Fusion-Approach (accessed on 18 December 2023).

## Supplementary Materials

The following supporting information can be downloaded at: https://www.mdpi.com/article/10.3390/jimaging10010028/s1. Figure S1. Comparison of enhanced images from Kvasir dataset; Figure S2. Comparison of enhanced images from Kvasir dataset; Figure S3. Comparison of enhanced images from Kvasir dataset; Figure S4. Comparison of enhanced images from Kvasir dataset; Figure S5. Comparison of enhanced images from Kvasir dataset; Figure S6. Comparison of enhanced images from Kvasir dataset; Figure S7. Comparison of enhanced images from Kvasir dataset; Figure S8. Comparison of enhanced images from Kvasir dataset; Figure S9. Comparison of enhanced images from Kvasir dataset; Figure S10. Comparison of enhanced images from Kvasir dataset.
