# Supplementary material for: Endoscopic Image Enhancement: Wavelet Transform and Guided Filter Decomposition-Based Fusion Approach
Source: J Imaging. 2024 Jan 20;10(1):28. doi: 10.3390/jimaging10010028 (PMC10816908; doi:10.3390/jimaging10010028)
Supplement: Supplementary file 1 [file jimaging-10-00028-s001.zip › jimaging-2790012-supplementary.pptx]

## Slide 1
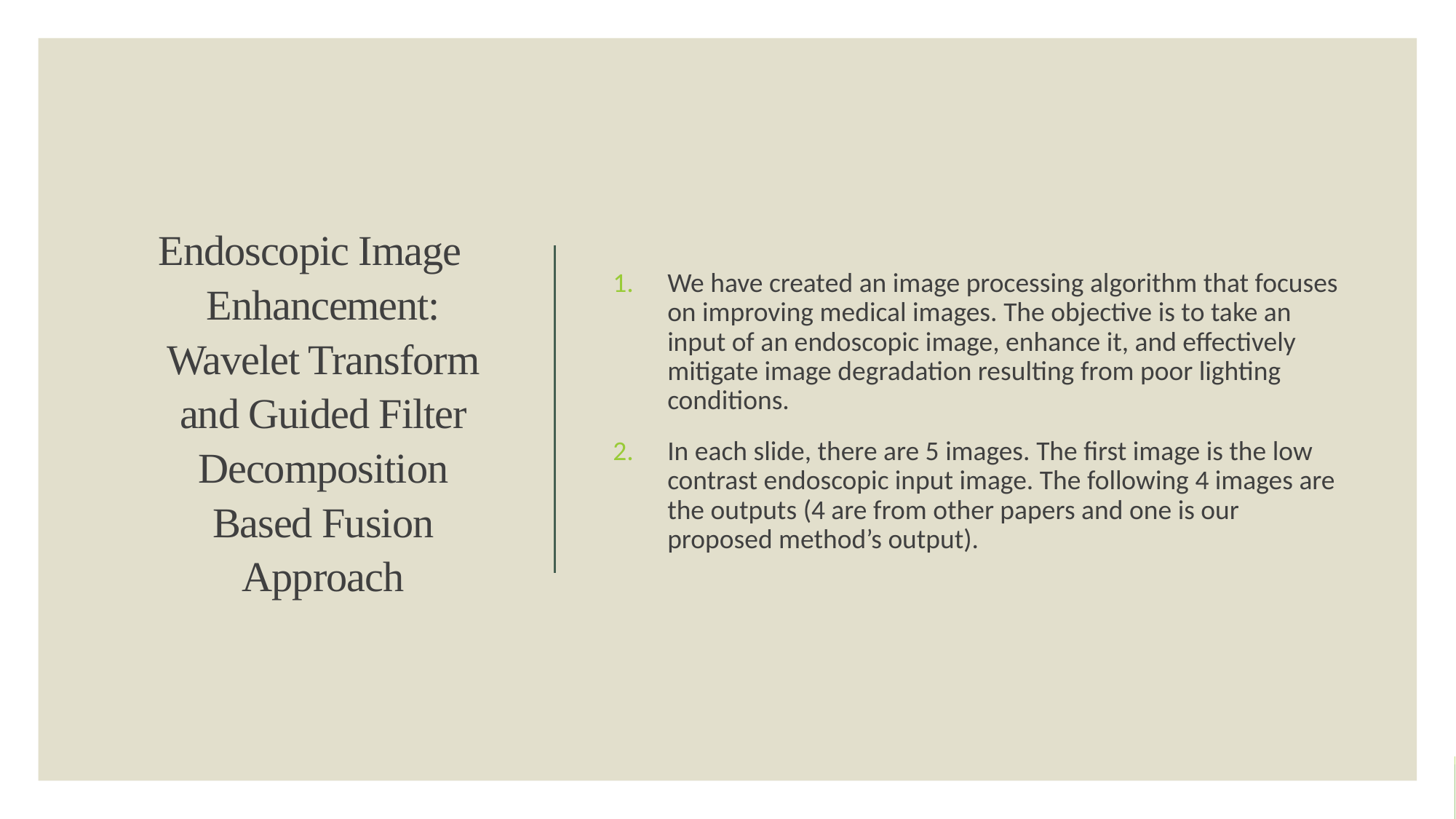

We have created an image processing algorithm that focuses on improving medical images. The objective is to take an input of an endoscopic image, enhance it, and effectively mitigate image degradation resulting from poor lighting conditions.
In each slide, there are 5 images. The first image is the low contrast endoscopic input image. The following 4 images are the outputs (4 are from other papers and one is our proposed method’s output).
Endoscopic Image Enhancement: Wavelet Transform and Guided Filter Decomposition Based Fusion Approach

## Slide 2
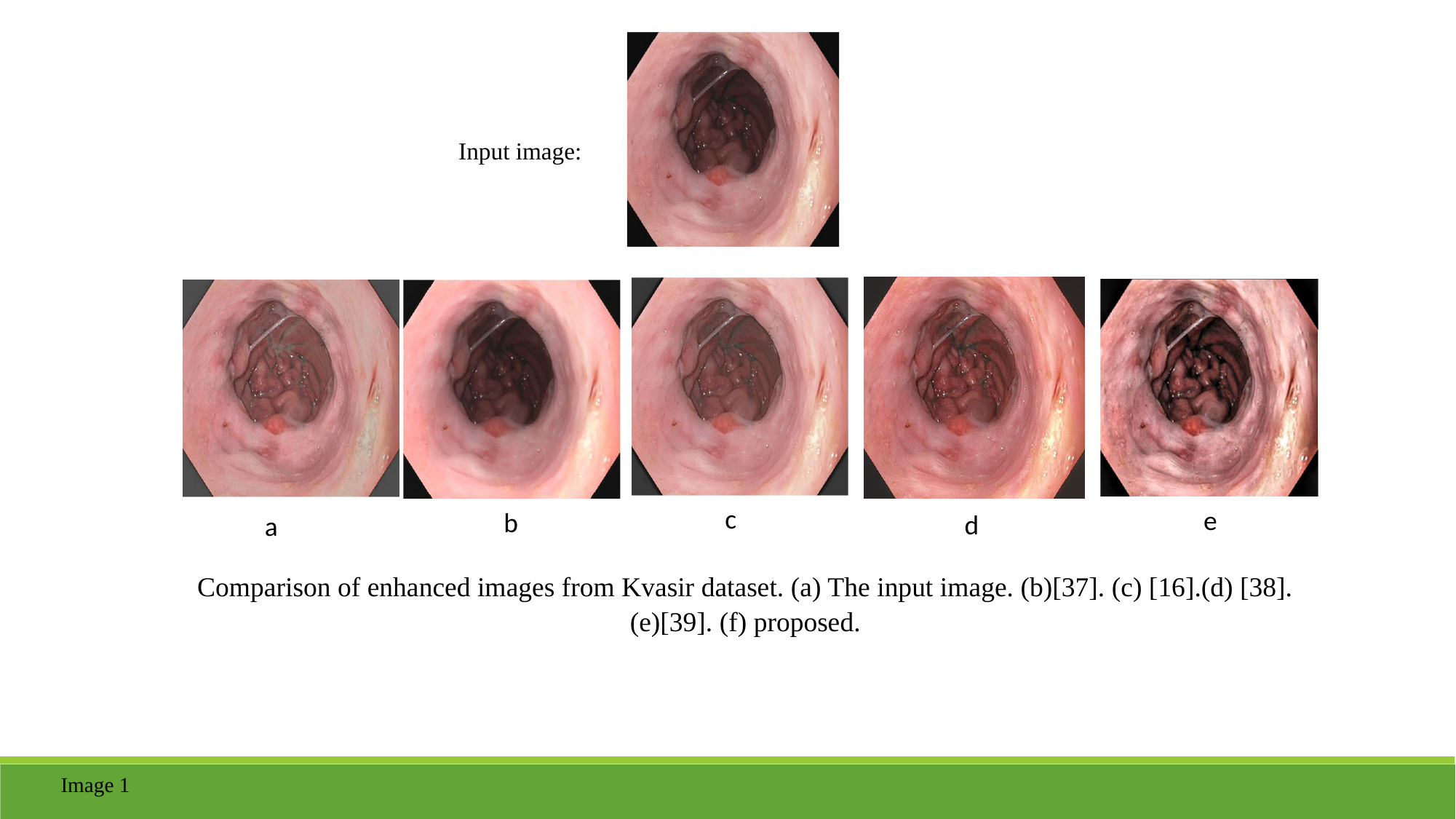

Input image:
c
e
b
d
a
Comparison of enhanced images from Kvasir dataset. (a) The input image. (b)[37]. (c) [16].(d) [38]. (e)[39]. (f) proposed.
Image 1

## Slide 3
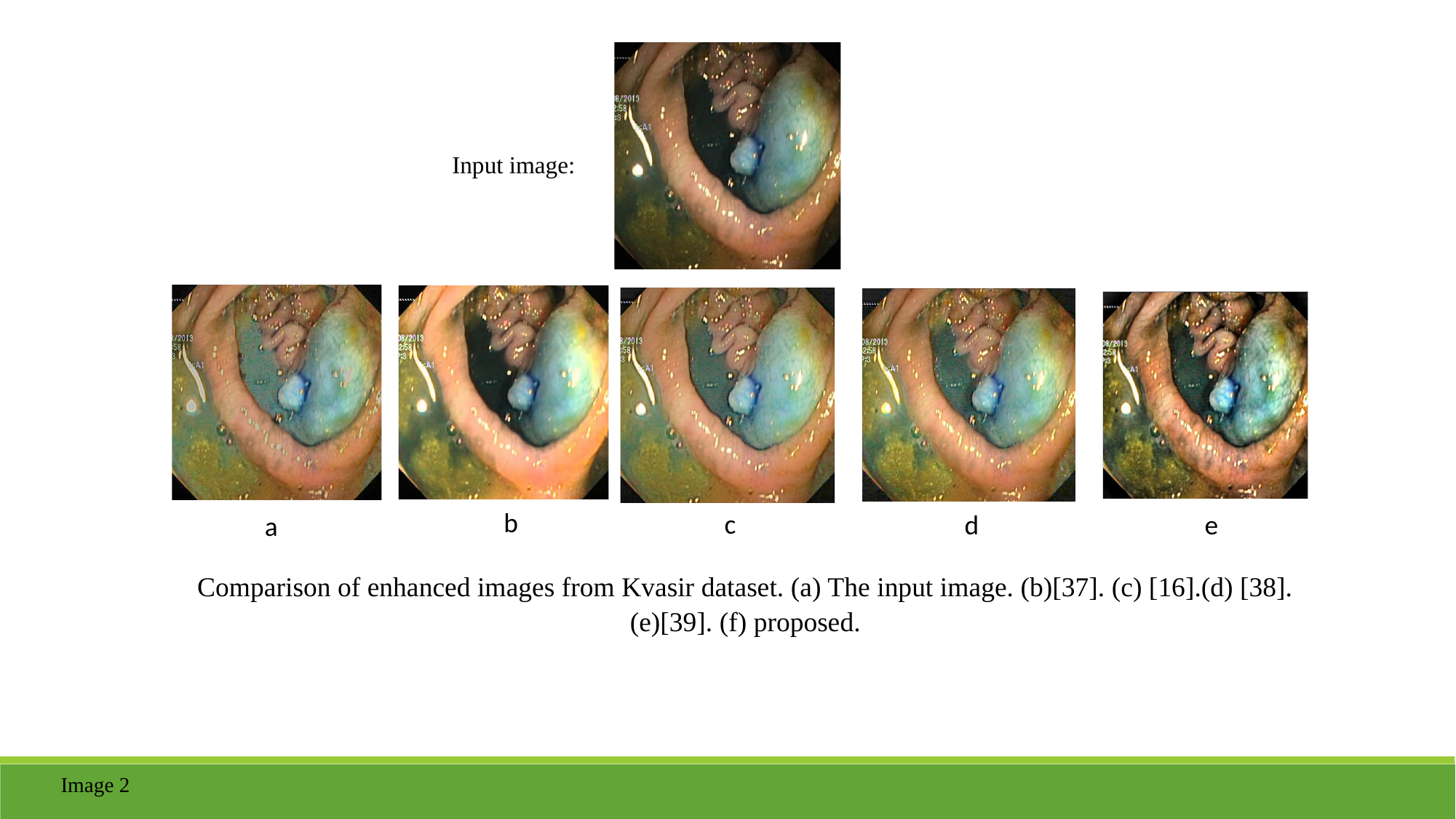

Input image:
b
c
d
e
a
Comparison of enhanced images from Kvasir dataset. (a) The input image. (b)[37]. (c) [16].(d) [38]. (e)[39]. (f) proposed.
Image 2

## Slide 4
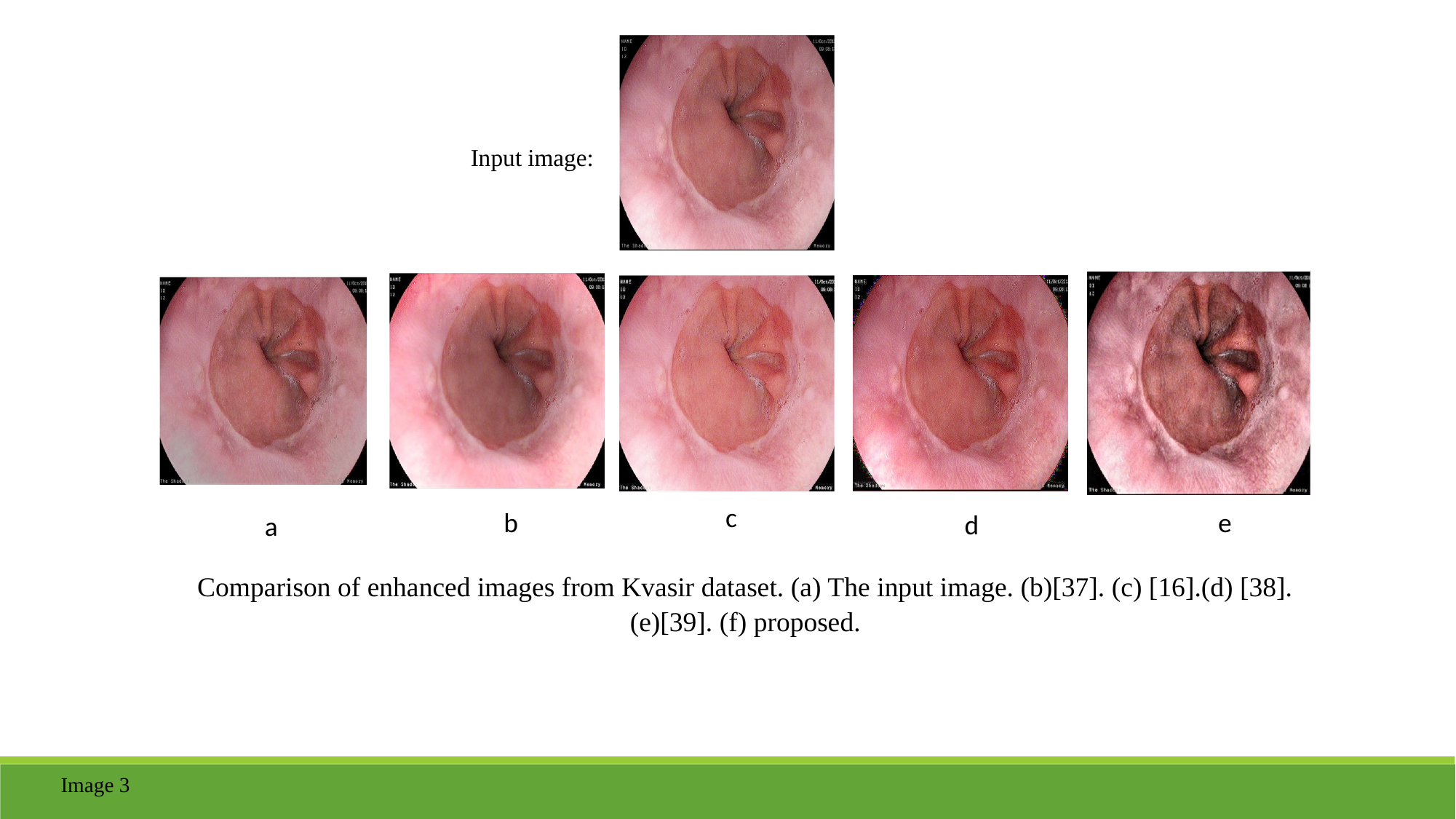

Input image:
c
e
b
d
a
Comparison of enhanced images from Kvasir dataset. (a) The input image. (b)[37]. (c) [16].(d) [38]. (e)[39]. (f) proposed.
Image 3

## Slide 5
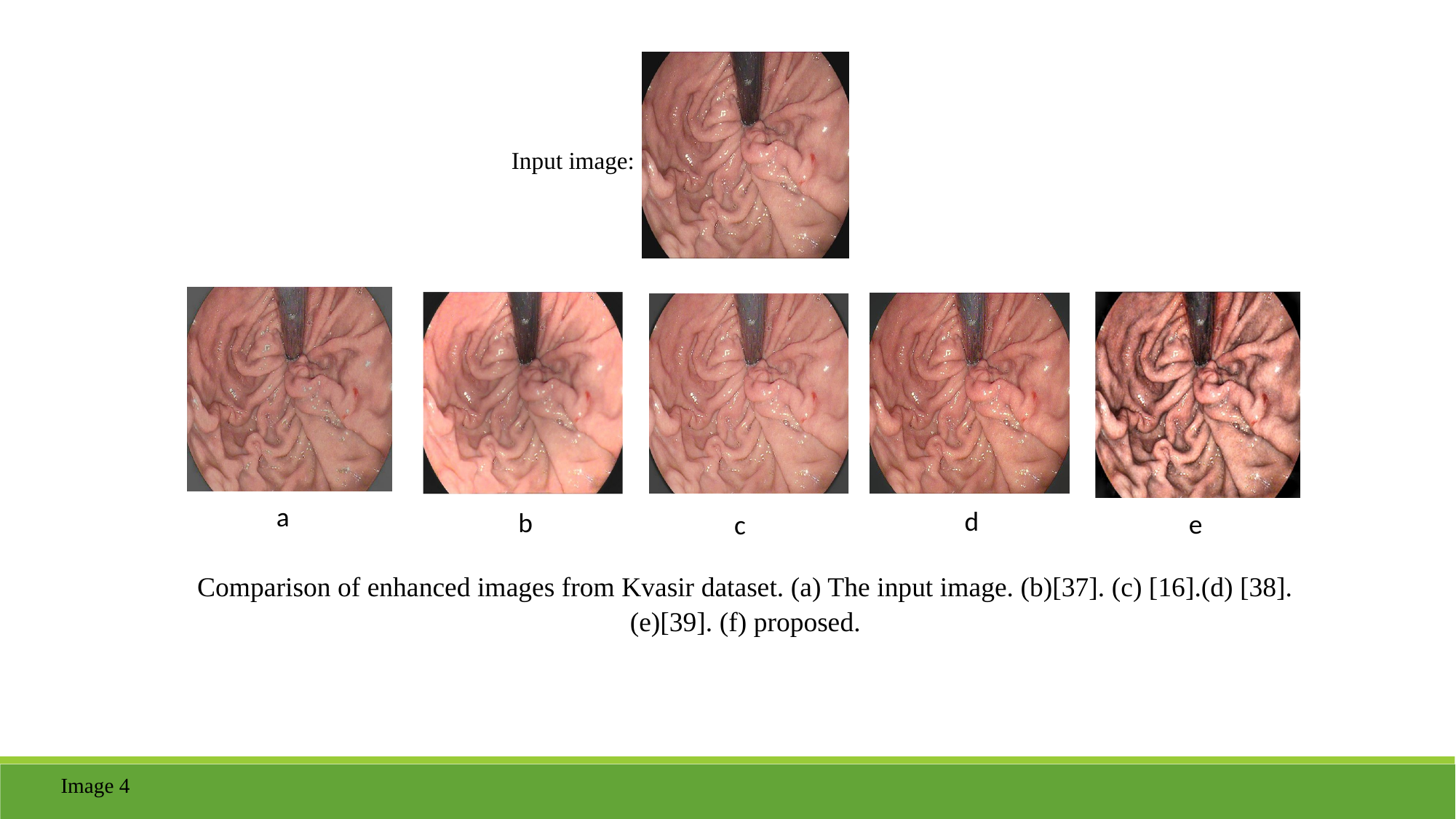

Input image:
a
d
b
e
c
Comparison of enhanced images from Kvasir dataset. (a) The input image. (b)[37]. (c) [16].(d) [38]. (e)[39]. (f) proposed.
Image 4

## Slide 6
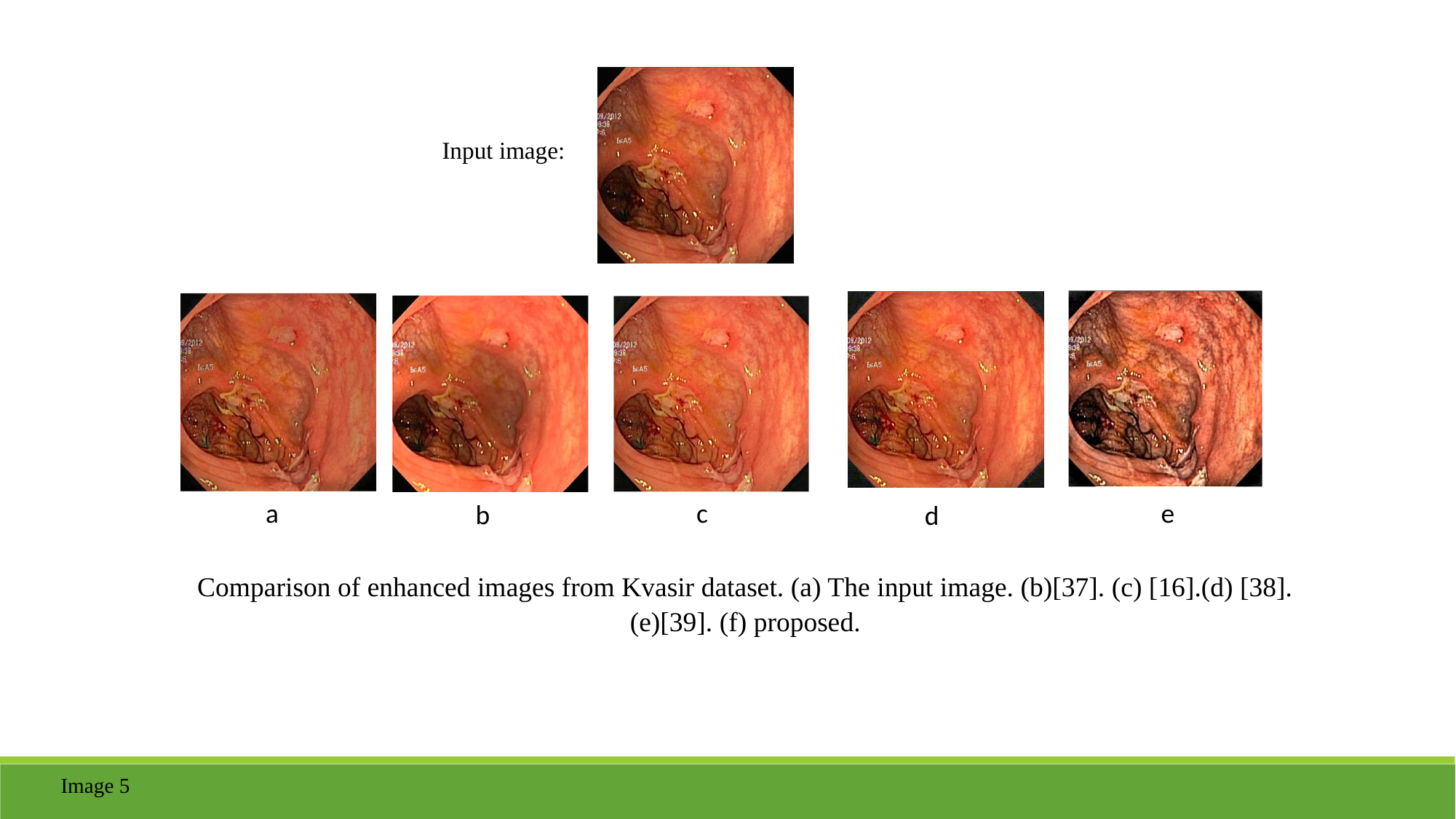

Input image:
a
c
e
b
d
Comparison of enhanced images from Kvasir dataset. (a) The input image. (b)[37]. (c) [16].(d) [38]. (e)[39]. (f) proposed.
Image 5

## Slide 7
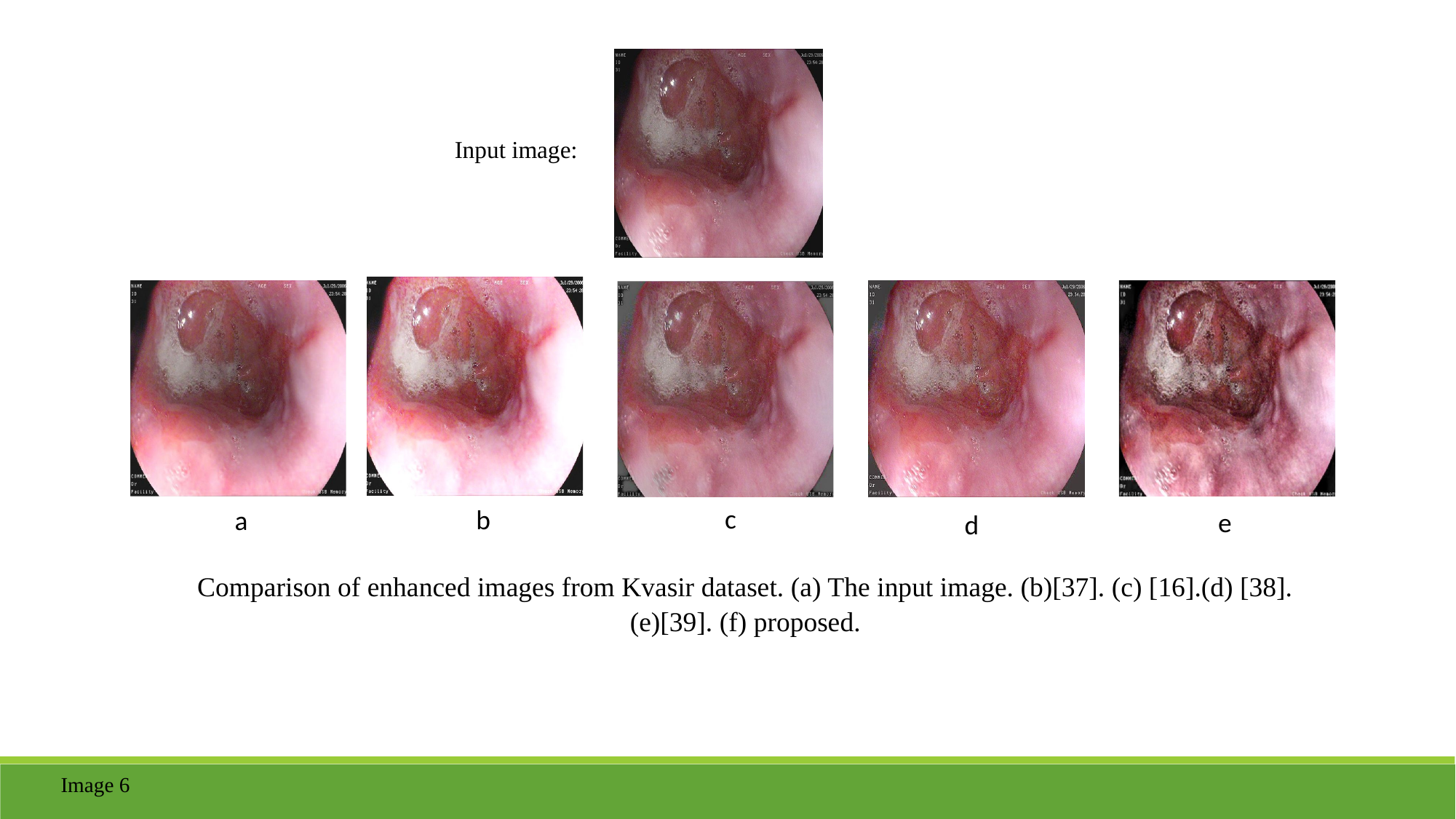

Input image:
c
b
a
e
d
Comparison of enhanced images from Kvasir dataset. (a) The input image. (b)[37]. (c) [16].(d) [38]. (e)[39]. (f) proposed.
Image 6

## Slide 8
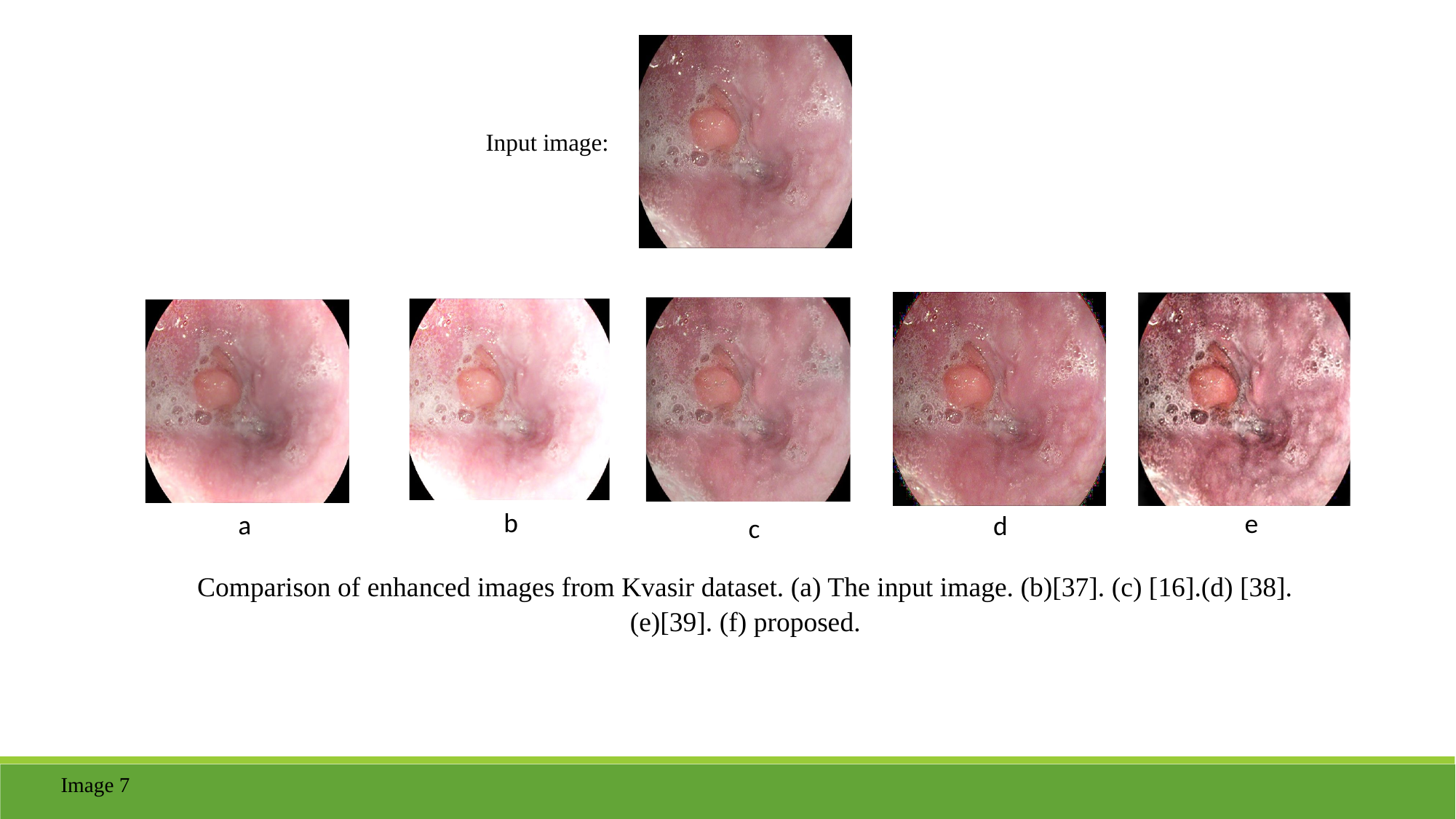

Input image:
b
e
a
d
c
Comparison of enhanced images from Kvasir dataset. (a) The input image. (b)[37]. (c) [16].(d) [38]. (e)[39]. (f) proposed.
Image 7

## Slide 9
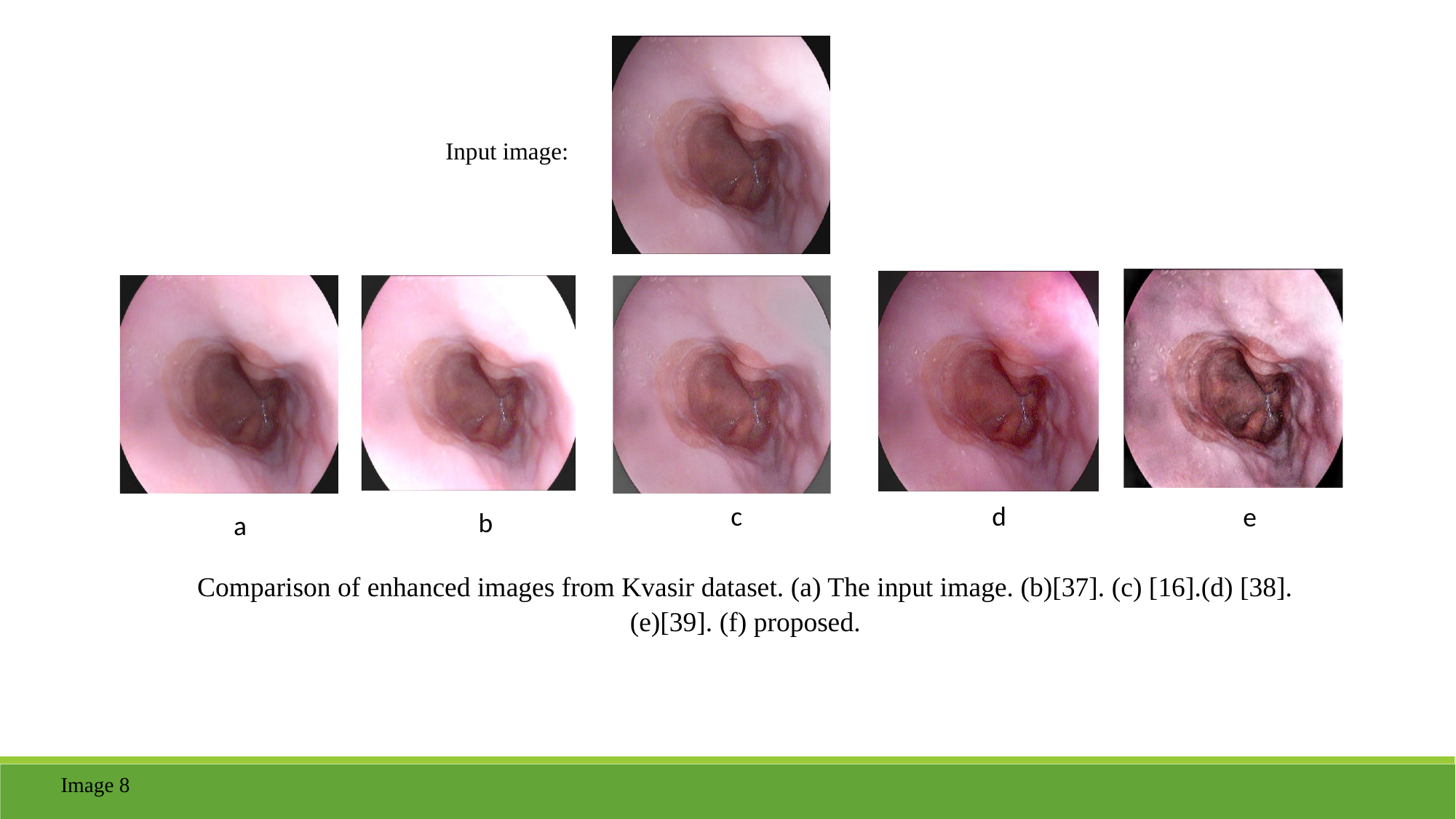

Input image:
c
d
e
b
a
Comparison of enhanced images from Kvasir dataset. (a) The input image. (b)[37]. (c) [16].(d) [38]. (e)[39]. (f) proposed.
Image 8

## Slide 10
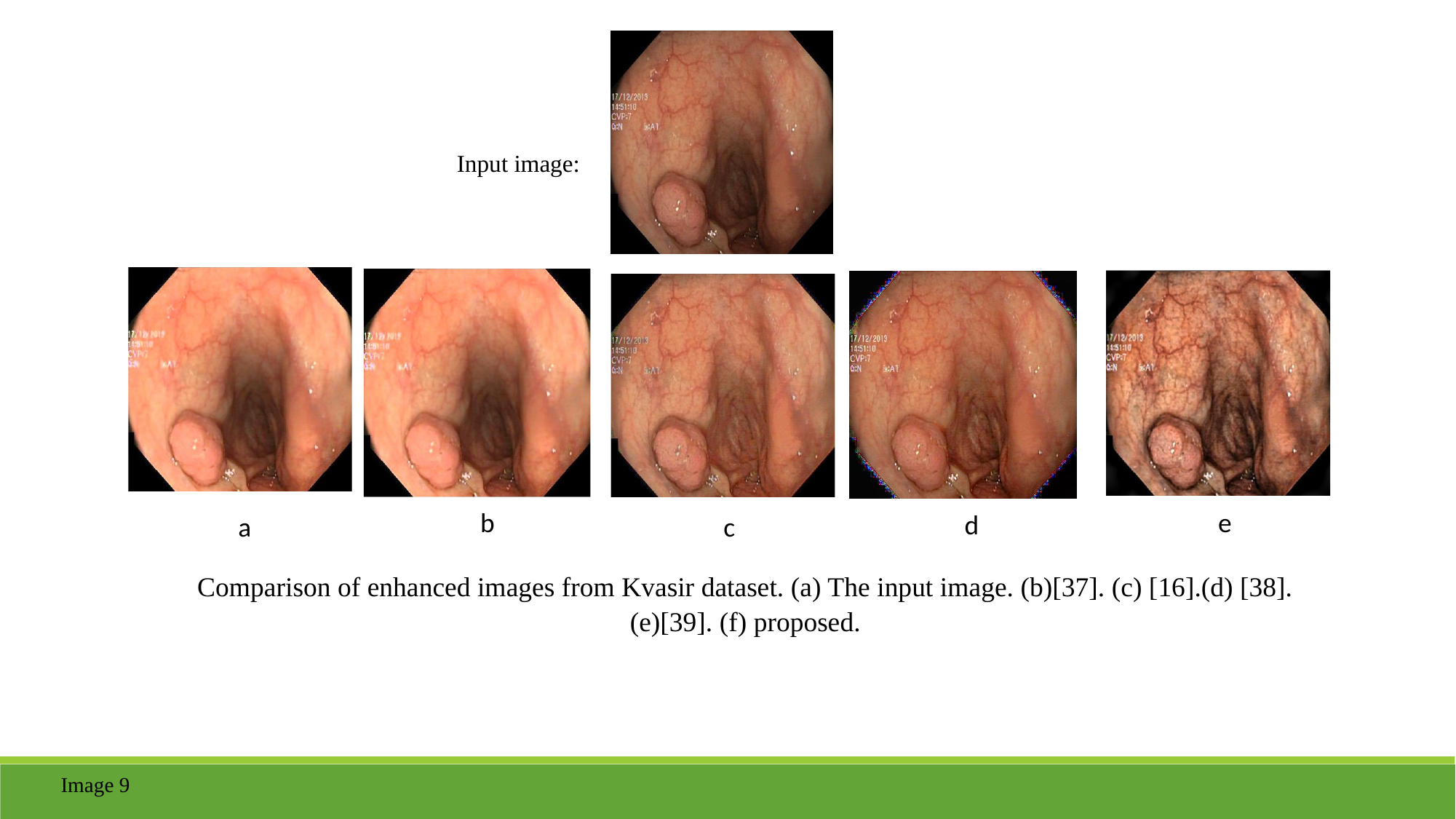

Input image:
e
b
d
a
c
Comparison of enhanced images from Kvasir dataset. (a) The input image. (b)[37]. (c) [16].(d) [38]. (e)[39]. (f) proposed.
Image 9

## Slide 11
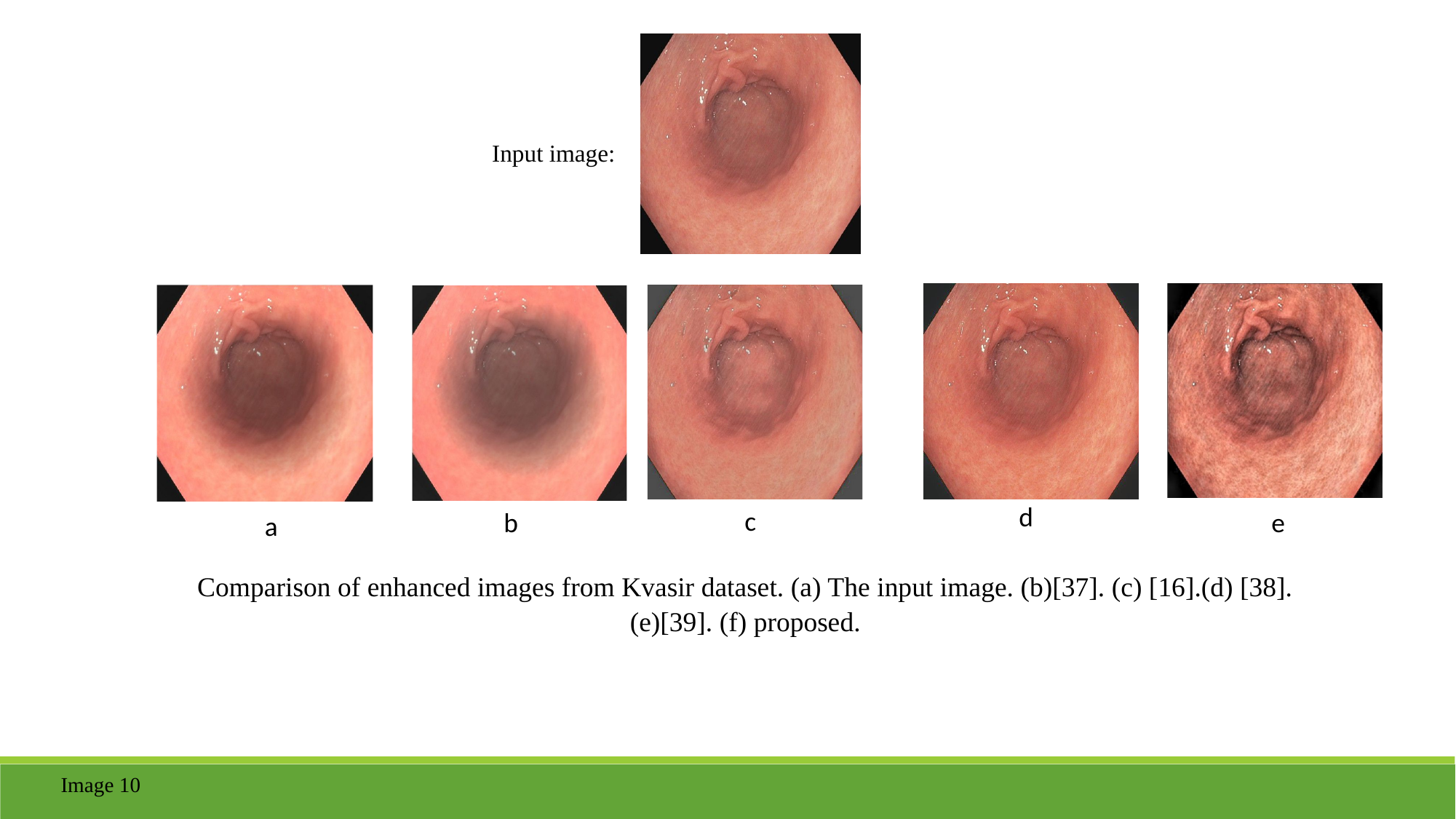

Input image:
d
c
b
e
a
Comparison of enhanced images from Kvasir dataset. (a) The input image. (b)[37]. (c) [16].(d) [38]. (e)[39]. (f) proposed.
Image 10

## Slide 12
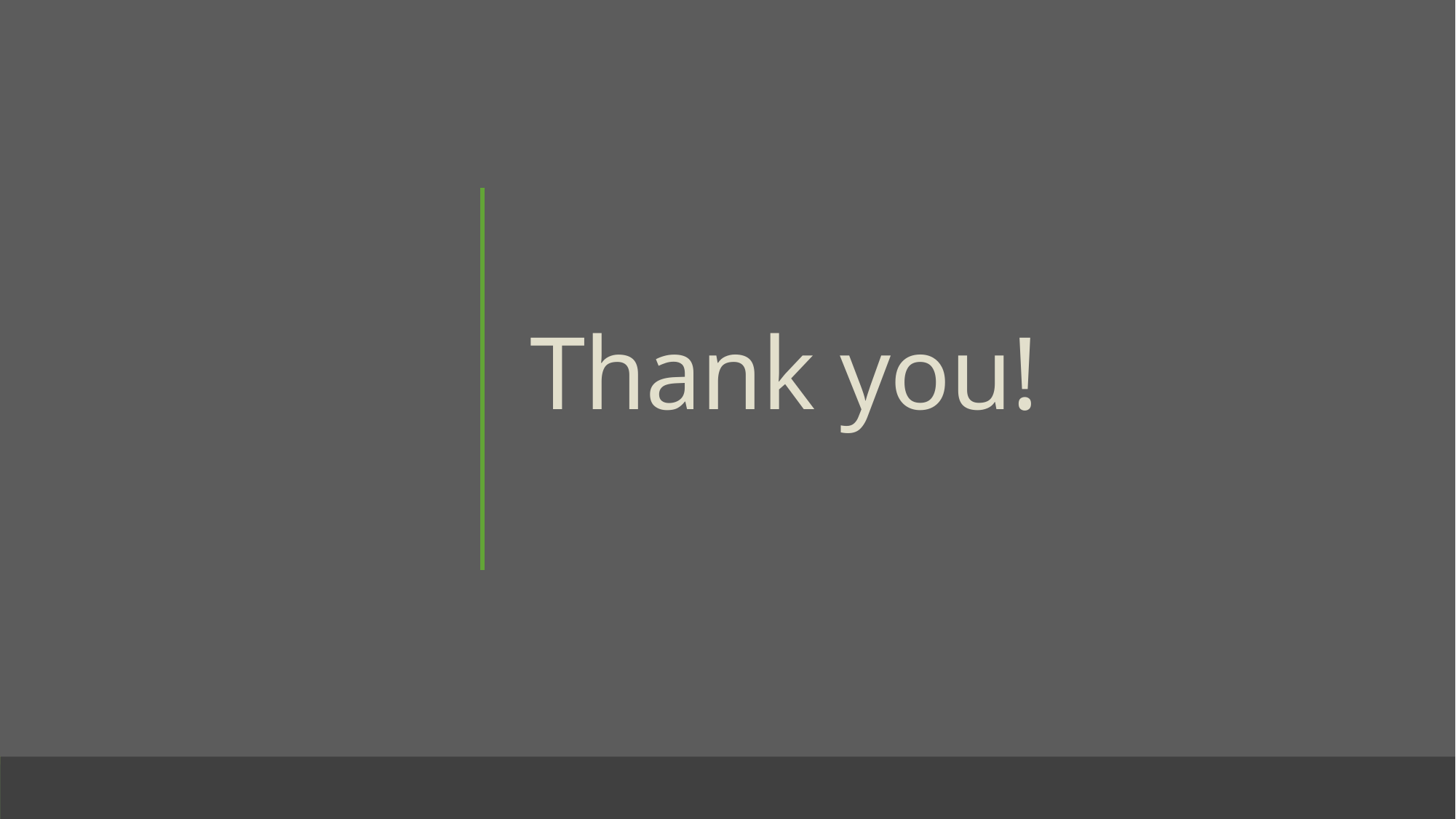

# Thank you!
